# Dimethyl Fumarate Alleviates NLRP3 Inflammasome Activation in Microglia and Sickness Behavior in LPS-Challenged Mice

**DOI:** 10.3389/fimmu.2021.737065

**Published:** 2021-11-10

**Authors:** Bora Tastan, Burak I. Arioz, Kemal Ugur Tufekci, Emre Tarakcioglu, Ceren Perihan Gonul, Kursad Genc, Sermin Genc

**Affiliations:** ^1^ Genc Laboratory, Izmir Biomedicine and Genome Center, Izmir, Turkey; ^2^ Izmir International Biomedicine and Genome Institute, Dokuz Eylul University, Izmir, Turkey; ^3^ Department of Healthcare Services, Vocational School of Health Services, Izmir Democracy University, Izmir, Turkey; ^4^ Department of Neuroscience, Health Sciences Institute, Dokuz Eylul University, Izmir, Turkey

**Keywords:** dimethyl fumarate (DMF), microglia, NLRP3 inflammasome, lipopolysaccharide (LPS), sickness behaviors, pyroptosis

## Abstract

NLRP3 inflammasome activation contributes to several pathogenic conditions, including lipopolysaccharide (LPS)-induced sickness behavior characterized by reduced mobility and depressive behaviors. Dimethyl fumarate (DMF) is an immunomodulatory and anti-oxidative molecule commonly used for the symptomatic treatment of multiple sclerosis and psoriasis. In this study, we investigated the potential use of DMF against microglial NLRP3 inflammasome activation both *in vitro* and *in vivo*. For *in vitro* studies, LPS- and ATP-stimulated N9 microglial cells were used to induce NLRP3 inflammasome activation. DMF’s effects on inflammasome markers, pyroptotic cell death, ROS formation, and Nrf2/NF-κB pathways were assessed. For *in vivo* studies, 12–14 weeks-old male BALB/c mice were treated with LPS, DMF + LPS and ML385 + DMF + LPS. Behavioral tests including open field, forced swim test, and tail suspension test were carried out to see changes in lipopolysaccharide-induced sickness behavior. Furthermore, NLRP3 and Caspase-1 expression in isolated microglia were determined by immunostaining. Here we demonstrated that DMF ameliorated LPS and ATP-induced NLRP3 inflammasome activation by reducing IL-1β, IL-18, caspase-1, and NLRP3 levels, reactive oxygen species formation and damage, and inhibiting pyroptotic cell death in N9 murine microglia *via* Nrf2/NF-κB pathways. DMF also improved LPS-induced sickness behavior in male mice and decreased caspase-1/NLRP3 levels *via* Nrf2 activation. Additionally, we showed that DMF pretreatment decreased miR-146a and miR-155 both *in vivo* and *in vitro*. Our results proved the effectiveness of DMF on the amelioration of microglial NLRP3 inflammasome activation. We anticipate that this study will provide the foundation consideration for further studies aiming to suppress NLRP3 inflammasome activation associated with in many diseases and a better understanding of its underlying mechanisms.

## 1 Introduction

Neuroinflammation is a conserved response to all kinds of threats in the central nervous system (CNS) involving certain cell types like neurons, microglia, and astrocytes (1). In acute neuroinflammation, microglia are activated at once, and a rapid response occurs against the dangerous stimuli (2).

Lipopolysaccharide (LPS) -induced sickness behavior is a condition which exhibits depression-like symptoms such as fatigue, cognitive impairment, and limited physical activity (3). A previous study by Aubert et al. showed that LPS is adequate for inducing sickness behavior in mice (4). Numerous pro-inflammatory cytokines have been proven to be associated with sickness behavior; interleukin-1β (IL-1β), interleukin-6 (IL-6), and tumor necrosis factor-α (TNF-α) (5). One important activated pathway is the NLR Family Pyrin Domain Containing Protein 3 (NLRP3) inflammasome during acute neuroinflammation (6).

Inflammasomes, the primary mediators of inflammation, are multiprotein complexes, the activation of which results in the release of inflammatory cytokines and can induce pyroptotic cell death (7). Among the various inflammasomes, NLRP3 inflammasome is the most widely characterized, especially in the CNS (8, 9). The NLRP3 inflammasome complex consists of the pattern recognition receptor NLRP3, an adaptor protein called apoptosis-associated speck-like protein (ASC), and the catalytic enzyme caspase-1 in its inactive form. NLRP3 inflammasome complex is activated in two-steps: “priming” and “activation”. The priming step involves a danger signal that causes an upregulation in the transcription of NLRP3 and pro-IL-1β and prepares cells to respond to NLRP3 stimulants (10). The most common priming signal is the activation of toll-like receptor 4 (TLR4) by the bacterial product LPS. The presence of LPS causes translocation of the inflammatory transcription factor Nuclear Factor Kappa B (NF-κB) into the nucleus, which upregulates the messenger RNA (mRNA) levels of NLRP3 and pro−IL−1β (11, 12). Once there is a sufficient amount of expressed NLRP3 in the cell, multiple stimuli can act as the second “activation” signal. Typical signals include pore-forming crystals, extracellular adenosine 5’-triphosphate (ATP), lysosomal damage, and mitochondrial reactive oxygen species (ROS) (11, 13–15). Upon activation, the NLRP3 inflammasome complex leads to the maturation of the pro-inflammatory cytokines pro-IL-1β and pro-IL-18. Inflammasome activation may result to further cleavage of Gasdermin D (GSDMD), a pore-forming protein. The formation of pores on the cell membrane results in pyroptotic cell death (16), characterized by cell-swelling, secretion of pro-inflammatory molecules, and the propagation of immune response in the CNS (17). Further investigations are required to develop a comprehensive understanding of these dynamic processes and better therapeutic strategies for disorders associated with NLRP3 inflammasome.

Dimethyl Fumarate (DMF) is a drug approved by the Food and Drug Administration (FDA) for Relapsing-Remitting Multiple Sclerosis (RRMS) since the early 2010s (18). Two different clinical trials (DEFINE and CONFIRM) demonstrated that DMF intake could lead to a decline in brain lesions and decreased relapse rates in RRMS patients (19, 20). *In vitro* and *in vivo* studies have proven that DMF possesses immunomodulatory (21), anti-inflammatory (22), anti-oxidative, and neuroprotective properties (23). The protective nature of DMF has been attributed to the activation of the Nuclear Factor Erythroid 2-related Factor 2 (Nrf2), as DMF is considered to be the most clinically developed Nrf2 activator (24). Once activated, Nrf2 upregulates anti-oxidative and cytoprotective Antioxidant Responsive Element (ARE)-containing genes (25). DMF also inhibits the pro-inflammatory pathway NF-κB, thus downregulating the expression of NLRP3 and pro-IL-1β and decreases inflammatory response (26). It has previously been shown that DMF suppresses NLRP3 inflammasome in THP-1 cells and the dextran sulfate sodium-induced experimental colitis model (27, 28). However, DMF’s protective effects and DMF’s mechanism of action on microglial NLRP3 inflammasome activation remain largely unexplored, since there are currently no relevant experimental study available.

In the present study, we aimed to investigate DMF’s effects on microglial NLRP3 inflammasome activation using both *in vitro* and *in vivo* models. We hypothesized that DMF would exert protective effects against NLRP3 inflammasome activation and inflammasome-induced pyroptosis in microglia. Herein, we demonstrated that DMF alleviated microglial NLRP3 inflammasome activation *via* modulation of NF-κB and Nrf2, and ameliorated pyroptotic cell death. Furthermore, our *in vivo* study on mice revealed that DMF intake restored LPS-induced sickness behavior. Taken together, these results indicate that DMF treatment prevented NLRP3 inflammasome activation in both *in vitro* and *in vivo* models.

## 2 Materials and Methods

### 2.1 Chemicals and Reagents

DMF, adenosine 5’-triphosphate disodium hydrate, LPS (LPS 055: B5), ML385, Fetal bovine serum (FBS), RPMI 1640 cell culture media, L-Glutamine, penicillin/streptomycin, phosphate-buffered saline (PBS), and trypsin/EDTA were purchased from Sigma-Aldrich (St. Louis, USA). Ultra-Pure LPS (LPS 0111: B4) were purchased from *In vivo*Gen (San Diego, USA). **Supplementary Table 1** contains all antibodies used in this research.

### 2.2 Cell Culture

Murine N9 microglia were provided by Dr. Paola Ricciardi-Castagnoli (Cellular Pharmacology Center, Milan, Italy) (29). Cells were sustained in RPMI 1640 containing 10% FBS and 2 mM L-Glutamine, 100 U/ml penicillin, and 100 µg/ml streptomycin at 37°C with 5% CO_2_. Treatments were introduced as follows unless a different experimental setup is implemented; DMF (10 µM) for 1 hour, LPS (1 μg/ml) for 4 hours, and ATP (5 mM) for 1 hour.

### 2.3 LDH Assay

The cytotoxicity of N9 microglia was determined by checking LDH release with Cytotoxicity Detection Kit^PLUS^ (Roche, Germany) by following the manufacturer’s protocol. Microplate reader Varioskan (Thermo, USA) was used to obtain the colorimetric change of cells at 492 nm with a reference wavelength of 630 nm. Cytotoxicity percentages were calculated as follows: Cytotoxicity = (OD Sample – OD Low Control) (OD Maximal Release –OD Low Control) x 100

### 2.4 Cell Viability Assay

According to the manufacturer’s protocol, N9 microglia viability was investigated with Cell Counting Kit-8 (Sigma Aldrich, St. Louis, USA). Microplate reader Varioskan (Thermo, USA) was used to determine the colorimetric change of cells at 450 nm with a reference wavelength of 630 nm. Cell viability percentages was calculated as the percentage of untreated cells.

### 2.5 ELISA

Secreted IL-1β and IL-18 levels were determined by IL-1β, and IL-18 sandwich enzyme-linked immunosorbent assay (ELISA) kits (Invitrogen, USA) according to the manufacturer’s instructions.

### 2.6 Real-Time qRT-PCR

Total RNA was extracted by using the Nucleospin RNA II Kit (Macherey-Nagel, Germany) according to the manufacturer’s protocol. Total RNA concentration was measured by the Nanodrop spectrophotometer (Thermo Fischer, USA), and 1 µg of RNA was used for reverse transcription with cDNA Revert-Aid First Strand cDNA Synthesis Kit (Thermo Scientific, USA) by following the manufacturer’s protocol. GoTaq^®^ qPCR Master Mix (Promega, USA) was used for performing quantitative real-time PCR with LightCycler ^®^ 480 Instrument II (Roche Life Science, USA) according to the manufacturer’s protocol.

For miRNA studies, RNA was isolated by the miRNeasy mini kit (Qiagen, Germany). miScript II RT Kit and QuantiTect SYBR Green PCR Kits (Qiagen, Germany) were used for cDNA synthesis and qPCR, respectively. All the primers utilized in experiments are listed in **Supplementary Table 2**. Relative expression levels were calculated with the 2^−ΔΔCt^ method.

### 2.7 Protein Extraction and Western Blot Analysis

Protein extraction and western blot analysis were carried out as described before (30). Briefly, RIPA lysis buffer (50mM Tris-HCL, pH 7.4, 150mM NaCl, 0.25% deoxycholic acid, 1% Nonidet P-40, 1mM EDTA) supplemented with protease and phosphatase inhibitor (Thermo Scientific, Massachusetts, USA) was used for total protein isolation from both cell lysate and supernatant. NE-PER, Nuclear and Cytoplasmic Extraction Reagents (Thermo Scientific, USA) were used to isolate nuclear and cytosolic fractions. For Western Blot, equal amounts of protein samples were run on a 8-15% SDS–PAGE gel depending on the protein of interest and then was transferred to the PVDF membrane. Depending on the antibodies used, the PVDF membrane was blocked with 5% BSA or 5% dry milk in TBS-T. The membrane was incubated with a primary antibody overnight at 4°C and a secondary antibody for 1 hour at room temperature. At the end of incubations, antigen-antibody complexes were screened with Luminata Forte Western HRP substrate (Merck Millipore, USA) using a densitometer (Vilber Lourmat Gel Imager System, CA). ImageJ software (National Institutes of Health, USA) was used for band density analysis (27). Results were normalized to β-actin and Lamin A/C for quantification or given as arbitrary unit (a.u.) for secreted proteins present in the supernatant.

### 2.8 Caspase-1 Activity Assay

Luminometric Caspase-Glo^®^ 1 Inflammasome Assay (Promega, USA) was used for measuring caspase-1 activity by following the manufacturer’s protocol. Centro XS3 lb 960 microplate luminometer (Berthold Technologies, Germany) was used to measure the luminescence of cells. Caspase-1 percentages were calculated as the percentage of untreated cells.

### 2.9 Propidium Iodide Staining for Pyroptosis Detection

Cells were treated with 50 μg/ml propidium iodide (PI) stain (Thermo Scientific, USA) in the last 15 minutes of ATP treatment. Fluorescent images were captured using the fluorescent microscopy system (Olympus IX-71, Japan). PI-positive and negative cells were counted by ImageJ software (National Institutes of Health, USA) (31). PI-positive cell percentages were calculated by comparing them to untreated cells.

### 2.10 Mitochondrial ROS Measurement With MitoSOX

Mitochondrial ROS (mtROS) was determined with the MitoSOX reagent. After treatment, microglia were treated with 5 μM MitoSOX (Invitrogen, USA) for 15 minutes at 37°C, and fluorescence absorbance values were measured at 530 nm with a reference wavelength of 590 nm with the microplate reader Varioskan (Thermo scientific, Massachusetts, USA). For immunofluorescence assay, cells were treated with 5 μM MitoSOX for 15 minutes at 37°C and 0.1 μM DAPI for 2 minutes at 37°C. Images were obtained under an inverted fluorescent microscope, Olympus IX-71 (Olympus, Japan).

### 2.11 Intracellular ROS Measurement With DCFDA

Total intracellular ROS was investigated with CM-H2DCFDA (Invitrogen, USA) according to the manufacturer’s instructions. The fluorometric measurements were done at 495 nm with a reference wavelength of 527 nm with a Varioskan Flash (Thermo Scientific, USA) microplate reader.

### 2.12 Mitochondria Membrane Potential Measurement

JC-1 (Thermo, USA) stain was used for investigating mitochondria membrane potential measurement of microglia according to the manufacturer’s instructions. FACS Canto II analyzer (Becton Dickinson, USA) using a 488 nm laser (Becton Dickinson, USA) was used for assessing the mitochondria membrane potential.

### 2.13 Animals and Experimental Design

All the animal experiments and animal care were strictly performed according to the Izmir International Biomedicine and Genome Institute Local Ethic Committee for Animal Experiments (IBG-AELEC), protocol number: 03/2017. For *in vivo* study, male BALB/c, aged 12–14 weeks, were used. All the animals were housed and maintained in IBG-Vivarium under controlled conditions (22 ± 2°C; 12 hours light/dark periods) with access to food and water *ad libitum*. In the experimental setup, mice were grouped randomly into four different groups: Control, LPS, DMF + LPS, and ML385 + DMF + LPS (n= 7 mice/group). Before the experimental procedures, all mice were weighed and controlled. Mice were intraperitoneally injected with ML385 (30 mg/kg) 24 hours before LPS injection, DMF (30 mg/kg) 1 hour before LPS injection, and LPS (5 mg/kg). After 24 hours from LPS injection, all animals proceeded with the clinical scoring, weighing, and behavioral tests. Animals were sacrificed by decapitation, and their brains were collected.

### 2.14 Clinical Evaluation and Behavioral Tests

To assess DMF’s effects on sickness behaviors, the following evaluations and behavioral tests were carried out.

#### 2.14.1 Clinical Scoring and Weighing

Sickness behaviors and bodyweight were scored and weighed by a blinded observer. To detect the change in body weight, animals were weighed before and after injections. Clinical scoring was carried out based on a 4-point scale, as described before (32). Accordingly, each symptom (lethargy, ptosis, and huddling) adds 1 point to the overall score. The absence of symptoms was accepted as 0.

#### 2.14.2 Open Field Test

OFT was carried out to assess sickness behavior. At the end of treatments, animals were gently put in the corner of plastic boxes (40 cm × 40 cm × 40 cm) whose floors were divided into equal squares. Their free movement was observed for 6 minutes. The number of squares crossed by animals was recorded and counted. The boxes were cleaned after each test with 70% ethanol to eliminate any sign of odor.

#### 2.14.3 Tail Suspension Test

After OFT, TST was performed. After treatments, each animal was hanged individually by adhesive tape separated by opaque fences. The tape was placed 1 cm from the tip of their tail. The behaviors of animals were monitored for a period of 6 minutes. Animals are considered immobile when they stay motionless for 5 seconds, and their immobility time was recorded.

#### 2.14.4 Forced Swim Test

Lastly, FST, a commonly used behavioral test for sickness behavior, was carried out as described previously (30). Briefly, an empty glass cylinder (30 cm height, 10 cm diameter) filled up to 25 cm was used for the tests, and the temperature of the water used was at 25 ± 1°C. The animals were placed into this cylinder and monitored for 6 minutes. The latency to the immobility in which animals float in the water without moving for at least 5 seconds was recorded for each animal. Water was changed after each FST test.

### 2.15 Microglia Isolation

Microglia isolation was performed as previously described (33). Briefly, animals were anesthetized and transcardially perfused with ice-cold PBS using a peristaltic pump. After removing meninges, brains were dissected and collected in cold buffer HBSS (Hanks’ balanced salt solution, Sigma, USA) containing 1% BSA and 1mM EDTA. Brains were chopped in a petri dish containing HBSS with the help of a razor blade and then filtered through a 70 µm cell strainer into a 50 ml falcon tube. Collected samples were centrifuged for 12 minutes at 300 x g and 10°C. The pellets were resuspended in 5 ml cold 37% Percoll in PBS, underlaid with 4 ml 70% Percoll and overlayed with 4 ml 30% Percoll in a 15 ml Falcon Tube. These falcons were centrifuged for 40 minutes at 600 x g and 10°C with 0 acceleration and 0 brakes. Finally, the cell layer was collected from the 70% and 37% Percoll interface, transferred into an equal volume of PBS containing 1% FBS, and centrifuged for 5 minutes, 400 x g, 4°C. The supernatant was discarded carefully, and 400 µl PBS was added to the tube, and cells were used in further experiments.

### 2.16 Immunofluorescence Staining

For immunostaining (ASC speck, NF-κB, and Nrf2 staining), cells were fixed with 4% paraformaldehyde (PFA) at 37°C for 15 minutes, then washed with PBS. Permeabilization and blocking were done using PBS containing 10% Goat Serum and 0.5% Triton-X-100 at 37°C for 30 minutes. Cells were incubated with primary antibody overnight, and the following day, the secondary antibody was incubated for 1 hour. Images were acquired with LSM 880 Confocal microscopy (Zeiss, Germany) or fluorescent microscope Olympus BX-61 (Olympus, Japan).

For primary microglia staining, isolated cells were centrifuged for 4 minutes, 1600 RPM at RT, fixed with 4% PFA for 15 minutes at 37°C, then washed 1-2 times with PBS, and permeabilized with PBS containing %0,1 Triton-x100 for 10 minutes at RT. After washing twice, cells were blocked with PBS containing 5% donkey serum for 30 minutes at RT and incubated for an overnight hour at 4°C with primary antibody IBA1 and NLRP3/Caspase-1 diluted in PBS. On the next day, the cells were rewashed with PBS twice and incubated with secondary antibody diluted in PBS for 1 hour at RT. In the last 20 minutes of incubation, Hoechst was added for counterstaining. In the end, the cells were washed PBS twice, and slides were mounted. Visualization of the slides was done using a fluorescent microscope Olympus BX-61 (Olympus, Japan).

All images were analyzed with ImageJ software (National Institutes of Health, USA). In the analysis of ASC speck images, ASC speck positive cells were counted, and data were given as the percentage of total cells. Image analysis was conducted bling to avoid experimental bias.

### 2.17 Statistical Analysis

Graphpad Prism 8.0 (Graphpad Software Inc., CA, USA) was used for all Statistical analyses. Shapiro–Wilk test was followed for the normality test. Accordingly, Mann–Whitney U-test and Student’s t-test were utilized for non-parametric and parametric data, respectively. For comparison of multiple groups with normal distribution, one-way ANOVA with Bonferroni multiple comparison corrections was used. On the other hand, for the comparisons with three or more group without normal distribution, Kruskal-Wallis test with Dunn’s multiple comparison was utilized. All data were provided as mean ± standard error of the mean. A p-value less than 0.05 (p<0.05) was considered as statistically significant in all experiments. All *in vitro* experiments were repeated three times, unless otherwise indicated.

## 3 Results

### 3.1 DMF Reduced NLRP3 Inflammasome Related Cytokines IL-1β and IL-18 at Both mRNA and Protein Levels in N9 Cells

**Figure 1:**
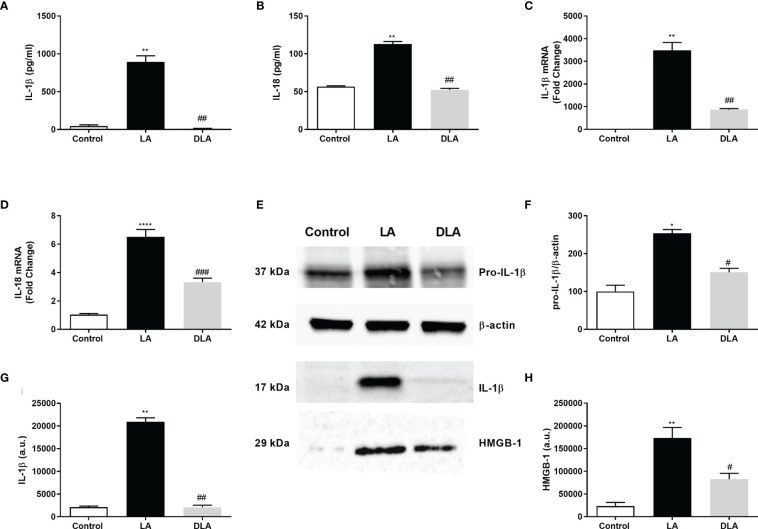
DMF suppressed inflammasome-induced secretion of pro-inflammatory cytokines and HMGB-1. N9 microglia were pretreated with DMF (10 µM) for 1hour, primed with UP-LPS (1000 ng/ml) for 4 hours, and followed by ATP (5 mM) for 1 hour. **(A, B)** Pretreatment with DMF decreased the protein and mRNA levels of IL-1β, IL-18, and HMGB-1. The protein levels of IL-1β, IL-18 were evaluated with ELISA. **(C, D)** mRNA levels of IL-1β and IL-18 were detected with qRT-PCR. **(E–H)** The expression of pro-IL-1β, IL-18, and their secretion along with HMGB-1 in supernatants were assessed by Western blot. Data are presented as mean ± S.E.M, n = 5. *p < 0.05, **p < 0.01, ****p<0,0001 compared to control and ^#^p < 0.05, ^##^p < 0.01, ^###^p<0,001 compared to LPS and ATP induced cells.

Ameliorative effects of DMF on secreted NLRP3 inflammasome-related cytokines IL-1β and IL-18 were tested with ELISA. We found that DMF significantly decreased the amounts of IL-1β (**Figure 1A**) and IL-18 (**Figure 1B**), which were induced by LPS and ATP treatment. Next, expression levels of IL-1β and IL-18 were evaluated with the RT-qPCR method. More than a 3-fold significant decline in IL-1β mRNA level (**Figure 1C**) and approximately 2-fold significant decline in IL-18 mRNA level (**Figure 1D**) were observed with DMF pretreatment. Furthermore, we observed that DMF significantly reduced both pro-IL-1β (**Figures 1E, F**) and secreted mature IL-1β protein levels (**Figures 1E, G**) against NLRP3 inflammasome activation. Additionally, we found that DMF significantly decreased High Mobility Group Box 1 (HMGB1) expression in LPS and ATP-induced microglia (**Figures 1E, H**).

### 3.2 DMF Decreased NLRP3, Caspase-1, and ASC, Which Forms the NLRP3 Protein Complex in N9 Cells

**Figure 2:**
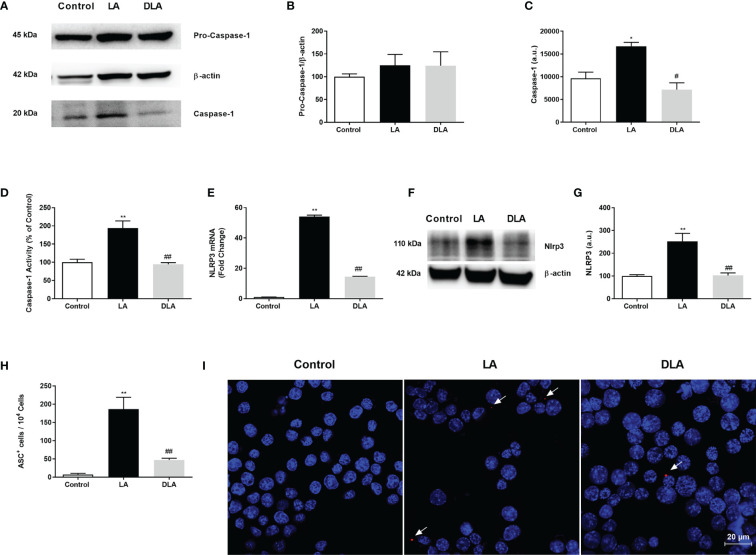
DMF decreased the expression of inflammasome complex proteins, prevented caspase-1 activity and ASC speck formation. N9 microglia were pretreated with DMF (10 µM) for 1h, primed with UP-LPS (1000 ng/ml) for 4 hours, and followed by ATP (5 mM) for 1 hour. Pretreatment with DMF led to decrease expression of NLRP3, inhibited cleavage of pro-caspase 1(p45) into caspase-1(p20), and prevented caspase-1 activity and ASC speck formation in microglia. **(A–C)** The expression of pro-caspase-1 and its cleavage were evaluated with Western blot. **(D)** Caspase-1 activity was measured with a luminometric assay. **(E)** mRNA expression of NLRP3 was measured with qRT-PCR. **(F, G)**. Protein expression of NLRP3 was detected by Western blot. **(H, I)** ASC speck formation was demonstrated by the IF method (Blue staining – Hoechst, Red staining – ASC). Data are presented as mean ± S.E.M, n = 5. *p < 0.05, **p < 0.01 compared to control and ^#^p < 0.05, ^##^p < 0.01 compared to LPS and ATP induced cells.

Next, we investigated DMF’s effect on the activated NLRP3 protein complex consisting of NLRP3, ASC adaptor protein, and Caspase-1. The intracellular level of p45 caspase-1 protein showed no significant difference in all three groups (**Figures 2A, B**). Active caspase-1 (p20) protein level significantly decreased in DMF pretreated cells compared to LPS and ATP-induced cells (**Figures 2A, C**). Additionally, Caspase-1 activity was evaluated and, there was a 2-fold increase with LPS and ATP treatment as it decreased 25% with DMF pretreatment (**Figure 2D**). Next, our investigations demonstrated that the DMF pretreated group showed an approximately 2-fold significant reduction in protein (**Figures 2E, F**) level and more than 2.5-fold significant reduction of the NLRP3 mRNA level (**Figure 2G**) as compared to the LPS and ATP treated group. Adaptor protein ASC is critically essential for the assembly of the NLRP3 inflammasome complex. Immunofluorescence staining results indicated that DMF diminished ASC speck formation induced by LPS and ATP administration (**Figures 2H, I**).

### 3.3 DMF Protected Against Pyroptotic Cell Death and Prevented Cleavage of GSDMD in N9 Cells

**Figure 3:**
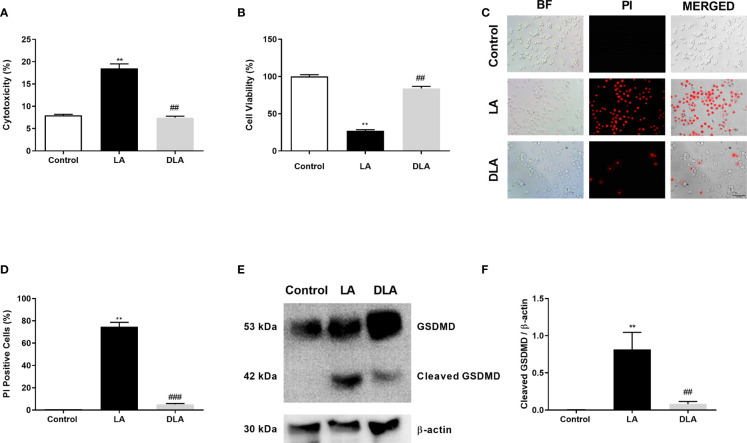
DMF attenuated GSDMD cleavage and subsequent pyroptotic cell death. N9 microglia were pretreated with DMF (10 μM) for 1h, primed with UP-LPS (1000 ng/ml) for 4 hours, and followed by ATP (5 mM) for 1 hour. DMF pretreatment inhibited NLRP3 inflammasome-induced GSDMD cleavage and pyroptotic cell death in N9 microglia. **(A, C, D)** Changes in pyroptotic cell death were evaluated with the LDH assay and PI staining (Red staining – PI). **(B)** The effect of DMF on cell viability was measured with the CCK-8 assay. **(E, F)** GSDMD expression and its cleavage were detected with Western blot. Data are presented as mean ± S.E.M, n = 5. **p < 0.01 compared to control and ^##^p < 0.01, ^###^p < 0.01 compared to LPS and ATP induced cells.

We further checked the effects of DMF on pyroptotic cell death stimulated by LPS and ATP administration. In the LDH assay (**Figure 3A**), we observed a significant decrease in cell death with DMF pretreatment compared to microglia with LPS and ATP treatment. Additionally, we revealed significant augmentation in microglia viability with DMF pretreatment (**Figure 3B**) We further carried out PI staining, a DNA intercalating dye that stains pyroptotic cells. We found a significant decrease in the number of pyroptotic cells with DMF pretreatment in PI staining (**Figures 3C, D**). Lastly, we evaluated the effect of DMF on GSDMD cleavage with western blotting and found a significant decline in cleaved GSDMD levels in DMF pretreated group against only the LPS and ATP treated group (**Figures 3E, F**).

### 3.4 DMF Prevented Mitochondrial and Total ROS Production and Enhanced Mitochondrial Membrane Potential *In Vitro*

**Figure 4:**
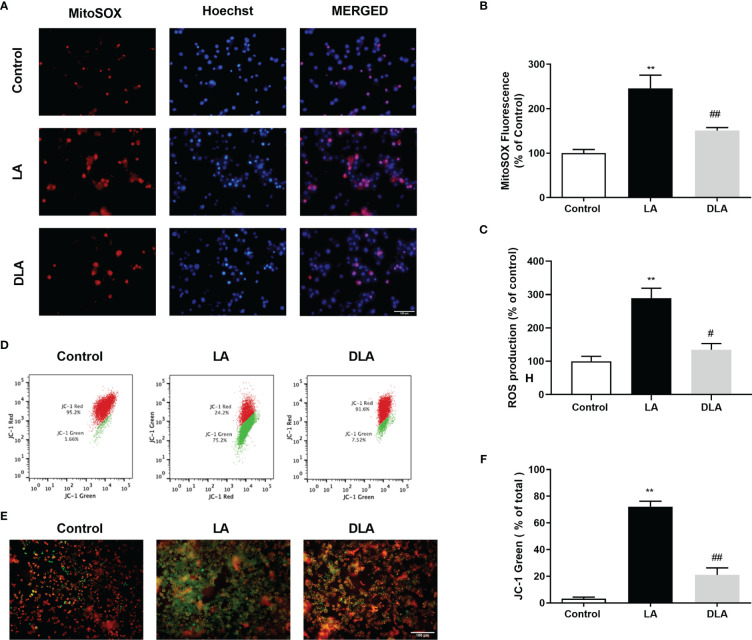
DMF decreased intracellular and mitochondrial ROS formation and restored mitochondrial membrane potential (ΔΨm). N9 microglia were pretreated with DMF (10 µM) for 1 hour, primed with UP-LPS (1000 ng/ml) for 4 hours, and followed by ATP (5 mM) for 1 hour. The anti-oxidative feature of DMF led to mitigated mitochondrial and intracellular ROS formation and balanced mitochondrial membrane polarization (ΔΨm). **(A, B)** The effects of DMF on mitochondrial ROS formation were evaluated with MitoSOX IF staining and MitoSOX fluorometric assay (Red staining- MitoSOX, Blue staining –Hoechst). **(C)** The intracellular ROS level was measured with a cellular ROS assay. **(D–F)** Changes in mitochondrial membrane potential were detected with JC-1 staining *via* Flow cytometry and IF staining (Red and Green Staining – JC-1). Data are presented as mean ± S.E.M, n = 5. **p < 0.01 compared to control and ^#^p < 0.05, ^##^p < 0.01 compared to LPS and ATP induced cells.

We studied mitochondrial ROS production with MitoSOX. Significantly elevated mtROS levels induced by LPS and ATP treatment were significantly reduced with DMF pretreatment (**Figures 4A, B**). After that, we investigated intracellular total ROS production of microglia with DCFDA. DMF pretreatment significantly alleviated, nearly 2.5-fold, total ROS production compared to the LPS, and ATP treated group (**Figure 4C**). Lastly, we checked the mitochondrial membrane potential with JC-1 staining. There was a significant decrease in the green ratio in DMF pretreated group compared only to the LPS, and ATP treated group *via* flow cytometry (**Figures 4D, F**); green signal indicates deteriorated mitochondrial membrane potential, whereas the red signal denotes healthy mitochondrial membrane potential. Furthermore, we confirmed the improvement of mitochondrial membrane potential with DMF pretreatment under a fluorescent microscope with JC-1 staining. We observed an increased green signal with LPS and ATP introduction and, conversely, an increased red signal and reduced green signal with DMF pretreatment (**Figure 4E**).

### 3.5 DMF Ameliorated NF-κB Activation and Reduced miR-155/146a Expression

**Figure 5:**
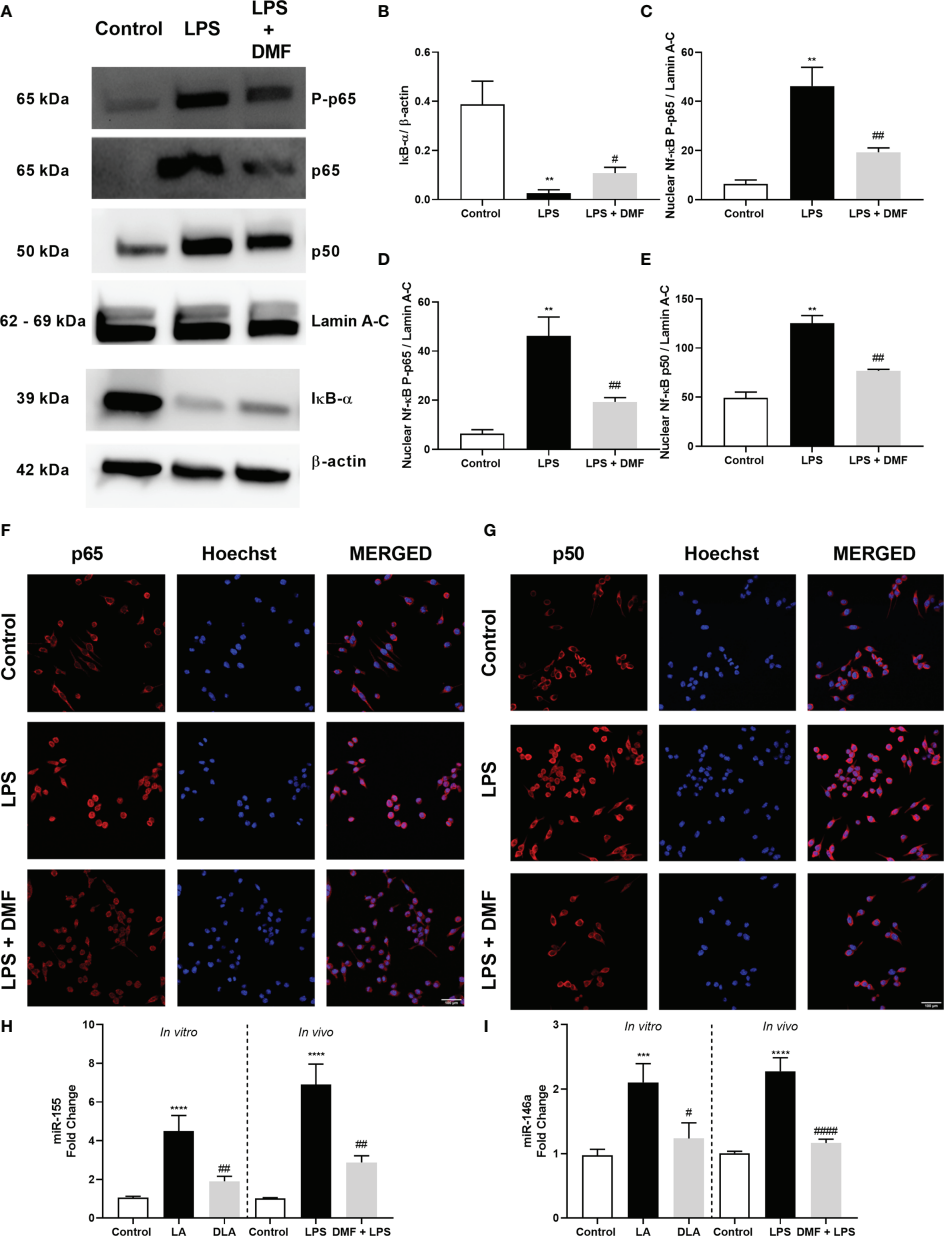
DMF prevented the translocation of NF-κB into the nucleus and altered miR-155 and miR-146a expression. N9 microglia were pretreated with DMF (10 µM) for 1hour, primed with UP-LPS (1000 ng/ml) for 1hour. DMF modulated NF-κB activity by inhibiting the translocation of p-p65, p65, and p50 subunits of NF-κB protein complex into the nucleus **(A).** The expression level of IκB-α **(B),** translocation of p-p65 **(C)**, p65 **(D),** and p50 **(E)** subunits of NF-κB were demonstrated with Western blot. Translocation of p65 **(F)** and p50 **(G)** subunits of NF-κB was demonstrated by IF staining (Red staining – NF-κB subunits, Blue staining – Hoechst). DMF also downregulated the expression of miR-155 and miR-146a. Expression levels of **(H)** miR-155 and **(I)** miR-146 in LPS+ATP treated N9 microglia and LPS challenged mice were measured with RT-qPCR. Data are presented as mean ± S.E.M, n = 5. **p < 0.01, ***p < 0.001, ****p < 0.0001 compared to control and ^#^p < 0.05, ^##^p < 0.01, ^###^p<0,001 compared to LPS-primed cells.

As the NF-κB pathway is the upstream pathway of the NLRP3 inflammasome pathway, we evaluated DMF’s effect on the NF-κB pathway with western blotting and immunostaining. Firstly, we found that DMF significantly increased the IκB-α protein level, the expression of which decreased by LPS treatment (**Figures 5A, B**). Next, we checked DMF’s effects on NF-κB subunits p65 and p50. We demonstrated that DMF significantly reduced nuclear p-p65 compared to the LPS treated group (**Figures 5A, C**). Furthermore, DMF pretreatment significantly reduced increased protein levels of nuclear p65 and p50 reduced with LPS treatment (**Figures 5A, D, E**). We also confirmed our result with immunostaining against the p65 and the p50 subunit of NF-κB as DMF decreased p50 and p65 translocation into the nucleus (**Figures 5F, G**). Lastly, we further checked the expression levels of miR-155 and miR-146a with the RT-qPCR method, as these miRNAs are known to act upstream of NF-κB and be related to inflammasome activation. We showed that DMF significantly decreases the expression levels of miR-155 (**Figure 5H**) and miR-146a (**Figure 5I**) in both *in vitro* and *in vivo* models of NLRP3 inflammasome activation.

### 3.6 DMF Induced Nrf2 Translocation and Modulated NLRP3 Inflammasome Activation *via* Nrf2 in N9 Cells and LPS-Challenged Mice

**Figure 6:**
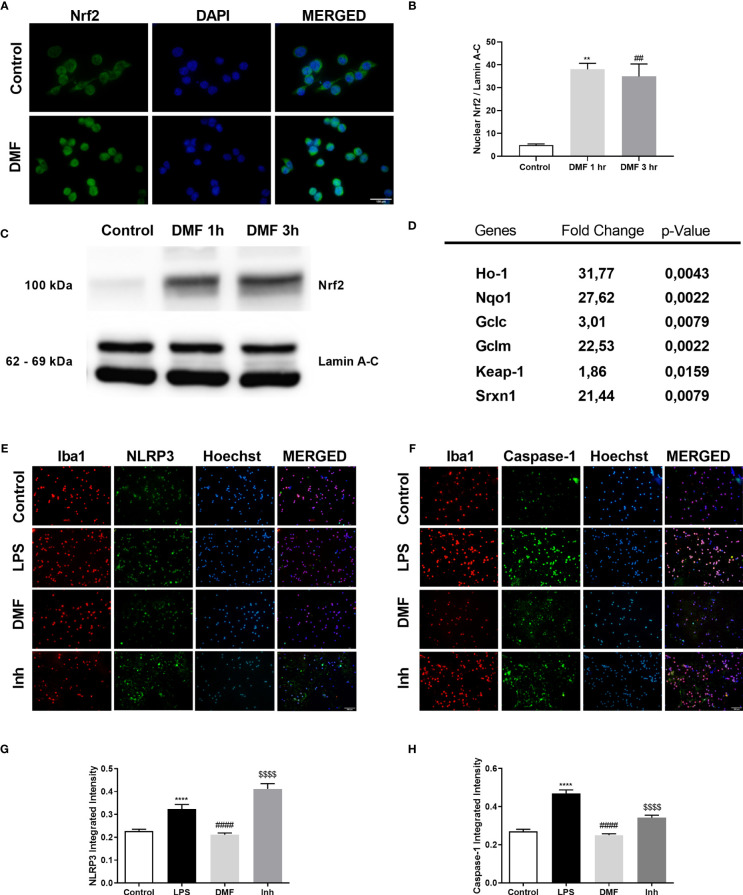
DMF induced Nrf2 activity, and inhibition of Nrf2 activity ameliorated the protective effects of DMF against microglial NLRP3 inflammasome activation. N9 microglia were treated with DMF (10 µM) for 1h or 3h. DMF induced the translocation of transcription factor Nrf2 into the nucleus and induced expression of Nrf2 target genes. **(A, C, D)** Translocation of Nrf2 was demonstrated with IF staining (Green staining – Nrf2, Blue staining – Hoechst) and Western blot. **(B)** mRNA levels of Nrf2 target genes were measured with qRT-PCR. **(E–H)** DMF reduced NLRP3 and Caspase-1 expression in microglia isolated from LPS-challenged mice (Red staining – Iba1, Green staining – Nlrp3/Caspase-1, Blue staining – Hoechst). Data are presented as mean ± S.E.M, n = 5. **p < 0.01, ****p < 0.0001 compared to control and ^##^p < 0.01, ^####^p < 0,0001 compared to LPS-challenged cells; ^$$$$^p < 0,0001 compared to DMF pretreated cells.

Given that DMF is a well-known activator of the Nrf2 signaling pathway, we further checked DMF’s effect on Nrf2 on microglia. First, we showed that DMF induced Nrf2 activation and led to the translocation of Nrf2 to the nucleus (**Figures 6A–C**). Next, we determined the expression levels of Nrf2 target genes, including Ho-1, Nqo1, Gclc, Gclm, Srxn1, and its inactivator Keap-1 by RT-qPCR. The results demonstrated that treatment with DMF increased the expression levels of the target genes and decreased Keap-1 expression (**Figure 6D**). We further confirmed our results with an *in vivo* study. In particular, microglia isolated from mice were stained against NLRP3 and Caspase-1. The results demonstrated that DMF decreased NLRP3 and Caspase-1 protein expression in those mice injected with DMF. However, ML385, a Nrf2 inhibitor, reversed this effect and increased the expression levels showing that Nrf2 mediates the protective effects of DMF (**Figures 6E**–**H**).

### 3.7 DMF Restored Sickness Behaviors in LPS-Challenged Mice

**Figure 7:**
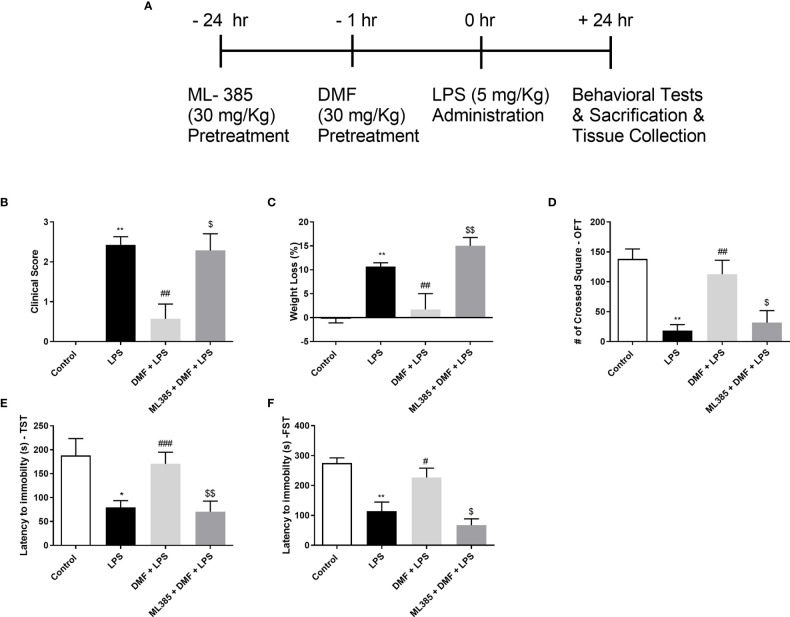
DMF alleviated sickness behaviors in LPS-challenged mice. DMF administration to LPS-challenged mice prevented weight loss and improved clinical and behavioral scores. **(A)** A schematic representation of the study design **(B)** Clinical evaluation of mice (n=7/group) and **(C)** Changes in the weight of mice. Assessment of sickness behaviors was conducted with OFT **(D)**, TST **(E),** and FST **(F).** Data are presented as mean ± S.E.M, n = 5. *p < 0,05, **p < 0.01 compared to control, ^#^p < 0.05, ^##^p < 0.01, ^###^p < 0,001 compared to LPS-challenged mice, ^$^p < 0,05, ^$$^p < 0,01 compared to DMF-administered mice.

In order to assess the effects of DMF on LPS-induced sickness behaviors, behavioral tests and clinical evaluation on mice were carried out (**Figure 7A**). In this regard, we conducted clinical scoring, weighed the mice, and performed OFT, FST, and TST. Results demonstrated that injection of LPS resulted in sickness behaviors in BALB/c mice while treatment with DMF reversed this situation (**Figure 7B**). On the other hand, pretreatment with Nrf2 inhibitor, ML385, reversed DMF’s protective effects on the LPS-induced sickness phenotype. We also weighed the animals before and after treatment. Results indicated that treatment with DMF decreased weight loss and inhibition of Nrf2 activation worsened this situation (**Figure 7C**). Next, we performed behavioral tests. In OFT, the animals treated with DMF crossed a significantly higher number of squares compared to LPS. However, the ones injected with ML385 traveled less in the open field (**Figure 7D**). Again, in TST and FST, DMF increased the latency to immobility. These effects also seem dependent on Nrf2 activation as the use of ML385 decreased the latency to immobility (**Figures 7E, F**). As a result, DMF significantly restored sickness behaviors, reversed clinical course, and prevented weight loss induced by LPS.

## 4 Discussion

In the present study, we proved that DMF ameliorated microglial NLRP3 inflammasome activation *via* the NF-κB/Nrf2 axis. The NLRP3 inflammasome activation is a fundamental part of the innate immune system, and its excessive activation leads to sterile inflammation (34). Mounting evidence suggests the involvement of inflammasome during LPS-induced sickness behavior. LPS reduces mobility and locomotion and increases pro-inflammatory cytokines like IL-1β and IFN-gamma in rats; these are characteristics of sickness behavior (35). Other cytokines like TNF-α and IL-6 were also increased by LPS administration in rodents (36). Our immunohistochemical analysis of microglia cells obtained from LPS-treated animals confirmed the presence of NLRP3 inflammasome activation. Our results (**Supplementary Table 3**) support that the activation of the NLRP3 inflammasome is involved in the pathogenesis of LPS-induced sickness behavior, and inhibition of NLRP3 inflammasome might represent a potential therapeutic strategy against inflammatory diseases.

In our study, we utilized the N9 microglial cell line in the *in vitro* assays. The N9 microglia were originally derived from the mouse brain, since previous studies confirmed that N9 shares standard features with primary microglia in morphology, immune response, and phagocytosis ability (29). Further studies also revealed that N9 cells can elicit the required response against inflammatory stimuli such as Amyloid-β, TNF-α, and IL-1β (37). Thus, N9 cell line has been extensively used in microglia research.

DMF is an FDA-approved Nrf2 activator and has been used safely in the treatment of RRMS. The neuroprotective (38), anti-oxidative (39), and anti-inflammatory (40) effects of DMF in the brain have been well documented in previous studies. In a recent study, where a rat model of chronic unpredictable mild stress was used, DMF restored sickness behaviors *via* the axis of MAPK/ERK1/2 and JNK pathways (41). However, to the best of our knowledge, there has been no study on the role of DMF in the regulation of NLRP3 inflammasome in the CNS. In previous studies, NLRP3 inflammasome suppression with DMF has been reported in diabetes-associated vascular complications (42) and dextran sulfate sodium-induced colitis (28). In the present study, we demonstrated that DMF exerted its protective effects both on the priming and activation step of NLRP3 inflammasome activation.

Oxidative stress and its leading cause, ROS formation, represent major risk factors for numerous neuropsychiatric disorders. Oxidative stress caused by mitochondrial damage and further mtROS formation also contribute to NLRP3 inflammasome activation in the CNS (43). It has been shown that mtROS activates the NLRP3 inflammasome in microglia (44, 45) through deubiquitination of NLRP3 and mitochondria released-DNA, a ligand of NLRP3 inflammasome (46). As an anti-oxidative agent, DMF ameliorates mitochondrial damage and ROS formation (47). These effects could be linked to the modulation of anti-oxidative Nrf2 and NF-κB signaling pathways.

DMF is a well-known and widely used Nrf2 activator approved for RRMS. Nrf2 is a transcription factor leading to the activation of protective pathways in the presence of ROS. Upon activation, Nrf2 unbounded from KEAP1, its inhibitor, and upregulated the expression of protective and anti-oxidant ARE genes, including HO-1, Nqo1, Srxn1, Gstp1, and Gclc (48). It has been demonstrated that DMF mediated these effects by oxidizing KEAP1 on cysteine 151 and translocating Nrf2 into the nucleus (49). Additionally, DMF was found to be the best Nrf2 activator among all other fumaric acid esters (50). The beneficial effects of DMF are also attributed to its ability to modulate the NF-κB pathway. It directly prevents the priming step of NLRP3 inflammasome activation by inhibiting the NF-κB pathway. Moreover, DMF indirectly inhibits NLRP3 inflammasome activation *via* the NF-κB pathway as the latter pathway is known to be a redox-sensitive regulator resulting to the expression of pro-oxidant enzymes, including NADPH oxidases NOX2 and COX2 (51). Lastly, the crosstalk between Nrf2 and NF- κB pathways might be increasing the potency of DMF’s anti-oxidative properties as upregulation of HO1 prevents the NF-κB pathway (52). Our study showed that DMF reversed mitochondrial damage and inhibited cellular ROS and mtROS formation, indicating DMF’s mitoprotective properties. DMF also inhibited the NF-κB pathway and downregulated expression of inflammatory miRNAs, miR-155 and miR-146a, suggesting that DMF induces differential expression of miRNAs, and therefore its protective nature might be substantially related to miRNAs. Furthermore, DMF induced the Nrf2 pathway and the use of ML385, specific Nrf2 inhibitor, confirmed DMF’s protective effects on NLRP3 inflammasome activation mediated by Nrf2. In the ML385-administered group, the reduced levels of NLRP3 and caspase-1 were reversed with DMF treatments. Therefore, our *in vivo* results indicated that DMF treatment had protective effects on LPS challenged mice *via* Nrf2 signaling.

Although we have demonstrated DMF’s protective effects on microglial NLRP3 inflammasome activation, our study has certain limitations. First, our *in vivo* study was conducted only in male mice. Sex-specific differences have been reported in microglia (53–55), and these differences may play a critical role in the alteration in the immune response. Thus, confirmation of our results in a mixed-sex study group in mice is required. Second, the focus of the current study is the microglial NLRP3 inflammasome. Although microglia are primary innate immune cells in the CNS, astrocytes express NLRP3 and other inflammasomes and participate in the immune response along with microglia (56, 57). Lastly, systemic inflammation caused by LPS administration in mice was not evaluated in the current study. Although we measured the clinical score and changes in body weight in LPS-challenged mice, other parameters and their implications for microglial activation were not examined.

## 5 Conclusion

**Figure 8:**
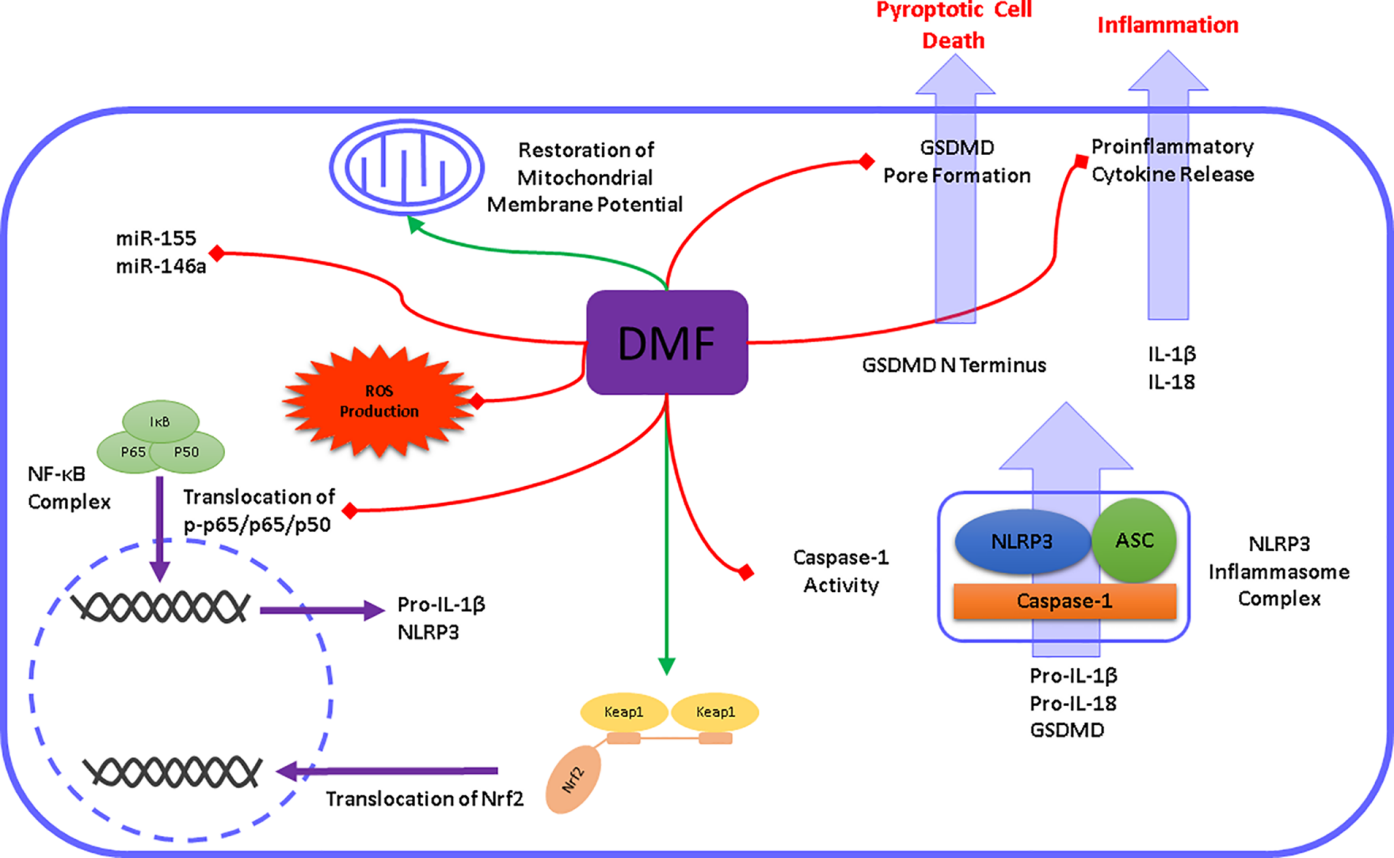
Schematic illustration of DMF modulating microglial NLRP3 inflammasome activation. DMF modulates the NLRP3 inflammasome signaling pathway at different levels. In the priming step, DMF prevents the translocation of NF-κB and subsequently downregulates the expression of NLRP3, IL-1β, and IL-18. Additionally, DMF promotes Nrf2 activation and upregulates the expression of Nrf2 target genes. Accordingly, DMF reduced the formation of cellular and mitochondrial ROS and restored the mitochondrial membrane potential. In the activation step, DMF inhibits caspase-1 cleavage and its activity. Subsequent cleavage of pro-inflammatory cytokines and pore-forming GSDMD was prevented by DMF.

Altogether, our findings demonstrated for the first time that DMF exerted protective properties on microglial NLRP3 inflammasome activation and subsequent pyroptotic cell death (**Figure 8**). As it is widely known that NLRP3 inflammasome activation contributes to numerous neurodegenerative and neuropsychiatric disorders, our study brings new insights into how DMF modulates NLRP3 inflammasome activation and lays the foundation for the development of novel strategies against conditions like LPS-induced sickness behavior.

## Data Availability Statement

The original contributions presented in the study are included in the article/**Supplementary Material**. Further inquiries can be directed to the corresponding author.

## Ethics Statement

The animal study was reviewed and approved by Izmir International Biomedicine and Genome Institute Local Ethic Committee for Animal Experiments.

## Author Contributions

BT, KG, and SG designed the study. BT, BIA, KUT, ET, CPG designed and performed the experiments. BT, BIA, KUT, ET, KG, and SG analyzed and interpreted the data. BT, BIA, KG, and SG wrote the manuscript. All authors contributed to the article and approved the submitted version.

## Funding

The study was funded by The Scientific and Technological Research Council of Turkey (TUBITAK, Project No: 215Z473) and Dokuz Eylul University Department of Scientific Research Projects (DEU-BAP, Project No: 2017.KB.SAG.039).

## Conflict of Interest

The authors declare that the research was conducted in the absence of any commercial or financial relationships that could be construed as a potential conflict of interest.

## Publisher’s Note

All claims expressed in this article are solely those of the authors and do not necessarily represent those of their affiliated organizations, or those of the publisher, the editors and the reviewers. Any product that may be evaluated in this article, or claim that may be made by its manufacturer, is not guaranteed or endorsed by the publisher.

## Abbreviations

ATP, Adenosine Triphosphate; ASC, Apoptosis-associated Speck-like Protein containing a CARD; CNS, Central Nervous System; DLA, Dimethyl Fumarate and LPS and ATP; DMF, Dimethyl Fumarate; ELISA, Enzyme-Linked Immunosorbent Assay; FBS, Fetal Bovine Serum; GSDMD, Gasdermin D; HMGB-1, High Mobility Group Box 1; IL, Interleukin; Inh, Inhibitor; LA, LPS and ATP; LPS, Lipopolysaccharide; mRNA, Messenger RNA; mtROS, Mitochondrial Reactive Oxygen Species; NF-κB, Nuclear Factor Kappa B; NLRP3, NLR Family Pyrin Domain Containing Protein 3; Nrf2, Nuclear Factor Erythroid 2-related Factor 2; PBS, Phosphate Buffer Saline; PFA, Paraformaldehyde; PI, Propidium Iodide; ROS, Reactive Oxygen Species; RRMS, Relapsing-Remitting Multiple Sclerosis; TNF-α, Tumor Necrosis Factor Alfa.
