# Supplementary material for: Dimethyl Fumarate Alleviates NLRP3 Inflammasome Activation in Microglia and Sickness Behavior in LPS-Challenged Mice
Source: Front Immunol. 2021 Nov 10;12:737065. doi: 10.3389/fimmu.2021.737065 (PMC8631454; doi:10.3389/fimmu.2021.737065)
Supplement: Supplementary file 1 [file Table_1.docx]

Supplementary Material

**Supplementary Table 1. Antibody list**

| Antibody | Company | Catalog # | Application | Dilution |
| --- | --- | --- | --- | --- |
| Anti-IL-1β | Abcam | Ab9722 | WB | 1:1000 |
| Anti-Caspase-1 | Abcam | Ab1872 | WB | 1:1000 |
| Anti-Caspase-1 | Adipogen | AG-20B-0042 | IF | 1:100 |
| Anti-NLRP3 | Adipogen | AG-20B-0014 | WB | 1:1000 |
| Anti-NLRP3 | Adipogen | AG-20B-0014 | IF | 1:100 |
| Anti-GSDMD | Abcam | Ab209845 | WB | 1:1000 |
| Anti-HMGB1 | Cell Signaling | Cst-3935S | WB | 1:1000 |
| Anti-NF-κB p65 | Santa Cruz | sc-372 | WB | 1:1000 |
| Anti-NF-κB p-p65 | Cell Signaling | Cst-3033 | WB | 1:1000 |
| Anti-NF-κB p50 | Cell Signaling | Cst-13586 | WB | 1:1000 |
| Anti-Nrf2 | Cell Signaling | Cst-12721 | WB | 1:1000 |
| Anti-Nrf2 | Cell Signaling | Cst-12721 | IF | 1:100 |
| Anti-Iba1 | Wako | 019-19741 | IF | 1:100 |
| Anti-ASC | Santa Cruz | sc-33958 | IF | 1:100 |
| Anti-β-actin | Abcam | Ab8227 | WB | 1:1000 |
| Anti-Lamin A/C | Santa Cruz | sc-20681 | WB | 1:1000 |
| Anti-Rabbit | Abcam | Ab1500073 | IF | 1:500 |
| Anti-Mouse | Abcam | Ab150107 | IF | 1:500 |
| Anti-Rabbit | Cell Signaling | Cst-7074 | WB | 1:2000 |
| Anti-Mouse | Cell Signaling | Cst-7076 | WB | 1:2000 |
