# Supplementary material for: Dimethyl Fumarate Alleviates NLRP3 Inflammasome Activation in Microglia and Sickness Behavior in LPS-Challenged Mice
Source: Front Immunol. 2021 Nov 10;12:737065. doi: 10.3389/fimmu.2021.737065 (PMC8631454; doi:10.3389/fimmu.2021.737065)
Supplement: Supplementary file 2 [file Table_2.docx]

Supplementary Material

**Supplementary Table 2. Primers list**

| Gene |  | Sequence (5′–3′) | Product Length |
| --- | --- | --- | --- |
| IL-1β | Forward | TTCTTTTCCTTCATCTTTGAAGAAG | 365 |
|  | Reverse | TCCATCTTCTTCTTTGGGTATTGTT |  |
| IL-18 | Forward | CTTTGGAAGCCTGCTATAATCC | 363 |
|  | Reverse | GGTCAAGAGGAAGTGATTTGGA |  |
| NLRP3 | Forward | TGCCTGTTCTTCCAGACTGGTGA | 143 |
|  | Reverse | CACAGCACCCTCATGCCCGG |  |
| GAPDH | Forward | ACCACAGTCCATGCCATCAC | 452 |
|  | Reverse | TCCACCACCCTGTTGCTGTA |  |
| NQO1 | Forward | TGGCCGAACACAAGAAGC | 262 |
|  | Reverse | TGAATCGGCCAGAGAATGAC |  |
| GCLM | Forward | TTGGCTTAGGCATCAGGGTG | 449 |
|  | Reverse | TGTGGTGAGTCCAACTGAGC |  |
| HO-1 | Forward | CACCCTGAGCTGCTGGTGGC | 523 |
|  | Reverse | CAGCCCCTGGGGGCCAGTAT |  |
| GCLC | Forward | GGCTCTCTGCACCATCAC | 459 |
|  | Reverse | TCTGACACGTAGCCTCGG |  |
| Keap1 | Forward | GGAATGAGTGGCGGATGATCA | 398 |
|  | Reverse | GCTTCAGCAGGTACAGTT |  |
| Srxn1 | Forward | GAAGAGGTATGGGGCTAC | 302 |
|  | Reverse | GCAGCCCCCAAAGGAATA |  |
| Nrf2 | Forward | TGGACGGGACTATTGAAGGCTG | 735 |
|  | Reverse | GCCGCCTTTTCAGTAGATGGAGG |  |
| miR-155-5p |  | UUAAUGCUAAUUGUGAUAGGGGU |  |
| miR-146a-5p |  | UGAGAACUGAAUUCCAUGGGUU |  |
