# Supplementary material for: Dimethyl Fumarate Alleviates NLRP3 Inflammasome Activation in Microglia and Sickness Behavior in LPS-Challenged Mice
Source: Front Immunol. 2021 Nov 10;12:737065. doi: 10.3389/fimmu.2021.737065 (PMC8631454; doi:10.3389/fimmu.2021.737065)
Supplement: Supplementary file 3 [file Table_3.docx]

Supplementary Material

**Supplementary Table 3. The highlights of the study**

| - DMF ameliorated microglial NLRP3 inflammasome activation and pyroptotic cell death. |
| --- |
| - DMF reduced cellular and mitochondrial ROS production and restored mitochondrial membrane potential. |
| - DMF modulated Nrf2 and NF-κB signaling pathways in protection against NLRP3 inflammasome activation |
| - DMF restored sickness behaviors in LPS-challenged mice in Nrf2 dependent manner. |
| - Dimethyl fumarate altered miR-155 and miR-146 expression in both *in vitro* and *in vivo*. |
